# Geniposide Ameliorates Liver Fibrosis Through Reducing Oxidative Stress and Inflammatory Respose, Inhibiting Apoptosis and Modulating Overall Metabolism

**DOI:** 10.3389/fphar.2021.772635

**Published:** 2021-11-24

**Authors:** Lu Yang, Liping Bi, Lulu Jin, Yuming Wang, Yuting Li, Zixuan Li, Wenju He, Huantian Cui, Jing Miao, Li Wang

**Affiliations:** ^1^ Tianjin University of Traditional Chinese Medicine, Tianjin, China; ^2^ Tianjin Second People’s Hospital, Tianjin, China; ^3^ First Teaching Hospital of Tianjin University of Traditional Chinese Medicine, Tianjin, China; ^4^ Yunnan Provincial Hospital of Traditional Chinese Medicine, Kunming, China; ^5^ Tianjin First Central Hospital, Tianjin, China; ^6^ Shandong Provincial Key Laboratory of Animal Cell and Developmental Biology, School of Life Sciences, Shandong University, Qingdao, China

**Keywords:** liver fibrosis, geniposide, untargeted metabolomic analysis, oxidative stress, inflammation, apoptosis

## Abstract

Liver fibrosis is a progressive liver damage condition caused by various factors and may progress toward liver cirrhosis, and even hepatocellular carcinoma. Many studies have found that the disfunction in metabolism could contribute to the development of liver fibrosis. Geniposide, derived from *Gardenia jasminoides* J. Ellis, has been demonstrated with therapeutic effects on liver fibrosis. However, the exact molecular mechanisms of such liver-protection remain largely unknown. The aim of this study was to explored the effect of geniposide on metabolic regulations in liver fibrosis. We used carbon tetrachloride (CCl_4_) to construct a mouse model of liver fibrosis and subsequently administered geniposide treatment. Therapeutic effects of geniposide on liver fibrosis were accessed through measuring the levels of hepatic enzymes in serum and the pathological changes in liver. We also investigated the effects of geniposide on inflammatory response, oxidative stress and apoptosis in liver. Furthermore, serum untargeted metabolomics were used to explore the metabolic regulatory mechanisms behind geniposide on liver fibrosis. Our results demonstrated that geniposide could reduce the levels of hepatic enzymes in serum and ameliorate the pathological changes in liver fibrosis mice. Geniposide enhanced the activities of superoxide dismutase (SOD) and glutathione peroxidase (GSH-Px) and decreased methane dicarboxylic aldehyde (MDA) levels in liver. Geniposide treatment also decreased the levels of interleukin (IL)-6, IL-1β, and tumor necrosis factor-alpha (TNF-a) in liver tissue homogenate. Terminal deoxynucleotidyl transferase-mediated dUTP-biotin nick end labeling assay (TUNEL) staining demonstrated that geniposide could reduce the apoptosis of hepatocytes. Geniposide increased the protein expression of B-cell lymphoma-2 (Bcl-2) and downregulated the protein expression of Bcl-2 Associated X (Bax), cleaved-Caspase 3, and cleaved-Caspase 9. Serum untargeted metabolomics analysis demonstrated that geniposide treatment improved the metabolic disorders including glycerophospholipid metabolism, arginine and proline metabolism, and arachidonic acid (AA) metabolism. In conclusion, our study demonstrated the protective effects of geniposide on liver fibrosis. We found that geniposide could treat liver fibrosis by inhibiting oxidative stress, reducing inflammatory response and apoptosis in the liver, and modulating glycerophospholipid, and arginine, proline, and AA metabolism processes.

## Introduction

Liver fibrosis can be caused by infection (hepatitis virus, parasites), hepatotoxic factors (drugs, industrial factors, alcohol), and environmental factors. It is a progressive disease characterized by the activation and regeneration of inflammatory cells and fibroblasts and the accumulation of extracellular matrices in the liver (Liang et al., 2021). In recent years, changes in dietary habits have increased the incidence of liver fibrosis. The World Health Organization (WHO) estimates that there are approximately 100 million patients with liver fibrosis and liver cirrhosis worldwide, resulting in more than one million deaths annually (Qiao et al., 2020). Over the long term, liver fibrosis can develop into liver cirrhosis and liver cancer, both of which can seriously threaten human health. Therefore, preventing the progression of liver fibrosis is important to reduce the incidence of both liver fibrosis and liver cancer.

Previous researches have demonstrated that etiologic treatment, such as long-term anti-virus therapy (entecavir and tenofovir), can inhibit and even reverse liver fibrosis (Liaw, 2013; Choi et al., 2021; Yang et al., 2021). However, etiologic treatment is still limited in its ability to treat liver fibrosis, since once the mechanism behind liver fibrosis has been activated, the disease continues to progress (Liaw, 2013). Therefore, it is important to develop novel anti-fibrotic drug candidates.

Metabolomics is an important tool for studying changes in endogenous metabolites and can elucidate the pathogenesis of diseases on a metabolic level (Cui et al., 2021). Many studies have identified changes in host metabolism during the progression of liver fibrosis (Zhang et al., 2018). Compared to healthy controls, serum carbohydrate, lipid metabolism, and amino acid metabolism all dramatically changed in liver fibrosis and liver cirrhosis patients due to decreased levels of leucine, isoleucine, valine, and myristic acid and increased concentrations of phenylalanine, tyrosine, tryptophan, and palmitic acid (Yoo et al., 2019) The serum of carbon tetrachloride (CCl_4_)-induced liver fibrosis mice exhibited higher levels of amino acids such as phenylalanine, tyrosine, glutamate, and lower levels of niacinamide and 3-hydroxyphenylacetate compared to mice without CCl_4_ treatment (Liang et al., 2016). Modulating host metabolism is one potential method of alleviating liver fibrosis. Amino acid metabolism, including lysine and tryptophan degradation, was altered in liver fibrosis patients who had their spleen removed (Yang et al., 2018). Geniposide could prevent CCl_4_-induced liver fibrosis by modulating glycolysis, fructose and mannose, glutathione, and sulfur metabolism (Song et al., 2017). Amarogentin has been found to have hepatoprotective effects by regulating the metabolism of amino acids and fatty acids (Zhang et al., 2018). Aqueous extracted from *Corydalis saxicola* Bunting could ameliorate the effects of liver fibrosis in rats by regulating amino acid metabolism, lipid metabolism, and pyruvate metabolism pathways (Liu et al., 2018).

**FIGURE 1:**
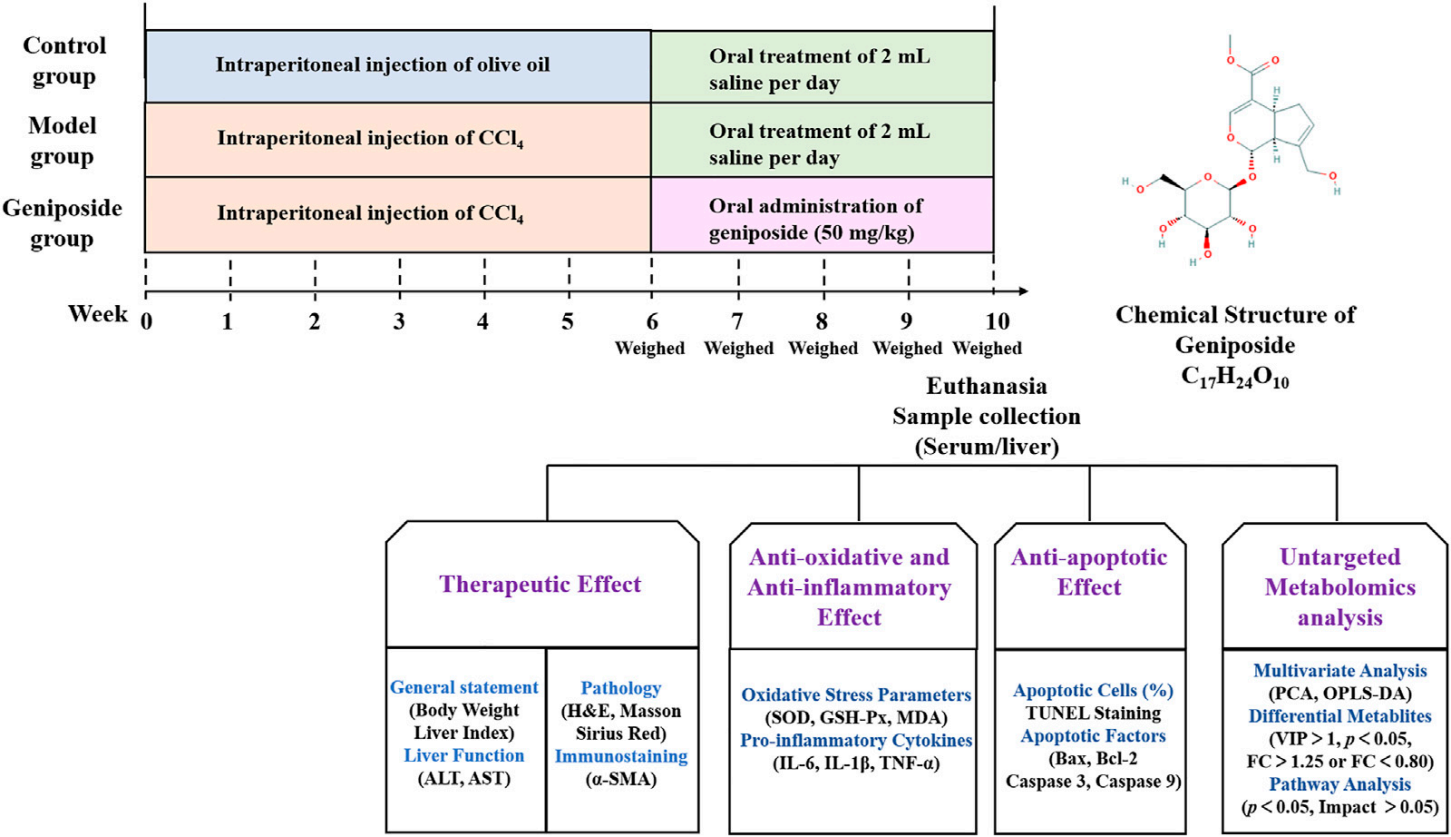
Overview of the experimental design for all groups.

Geniposide is an active ingredient extracted from *Gardenia jasminoides* J. Ellis and has multiple pharmacological effects, including anti-oxidative stress, anti-inflammatory properties, and anti-apoptotic effects (Yuan et al., 2020). It has been demonstrated that geniposide could downregulate the protein levels of alpha-smooth muscle actin (α-SMA), type I collagen alpha-1, Sonic hedgehog and Gli family zincfinger 1 in CCl_4_-induced liver fibrosis model mice (Lin et al., 2019). In addition, geniposide could reduce inflammatory response and increase bile acids secretion in a 3,5-diethoxycarbonyl-1,4-dihydrocollidine-induced sclerosing cholangitis model mice (Wen et al., 2021). *In vitro* study showed that geniposide could suppress TGF-β1-induced epithelial-mesenchymal transition in hepatocytes through inhibiting the activation of mitogen-activated protein kinase signaling pathway (Park et al., 2015). However, there are few studies assessing the metabolic changes in liver fibrosis model treated with geniposide. In this study, we constructed a liver fibrosis mouse model using CCl_4_, orally administered geniposide to these mice, and studied the effects of geniposide on oxidative stress, inflammation and apoptosis. We studied changes in serum metabolites following geniposide treatment using untargeted metabolomics (Figure 1).

## Materials and Methods

### Reagents

Geniposide (C_17_H_24_O_10_; molecular weight, 388.37 Da; virtue ≥98%) was obtained from Sichuan Weikeqi Biological Technology Co., Ltd. We obtained aspartate aminotransferase (AST), alanine aminotransferase (ALT), a bicinchoninic acid (BCA) protein quantitative kit, and superoxide dismutase (SOD), methane dicarboxylic aldehyde (MDA), and glutathione peroxidase (GSH-Px) assay kits from Nanjing Jiancheng Biological Engineering Institute. Mouse interleukin (IL)-6, IL-1β, and tumor necrosis factor-alpha (TNF-α) enzyme-linked immunosorbent assay (ELISA) kits were purchased from Shanghai BlueGene Biotech Co., Ltd. High-sig Enhanced chemiluminescence assay (ECL) western blotting substrates were obtained from Tanon Science and Technology Co., Ltd. Primary antibodies against mouse β-actin (20536-1-AP) were obtained from Proteintech Group, Inc. Terminal deoxynucleotidyl transferase-mediated dUTP-biotin nick end labeling assay (TUNEL) apoptosis detection kit, B-cell lymphoma-2 (Bcl-2, ab59348), Bcl-2 Associated X (Bax, ab32503), Caspase 3 (ab13847), and Caspase 9 (ab2786) were obtained from Abcam, Inc. The corresponding secondary antibodies were obtained from Abcam, Inc.

### Animals

C57BL/6 male mice were obtained from Beijing Huafukang Biotechnology Co. Ltd. The experimental animals were fed for 1 week prior to the experiment. All mice were kept in an environment at a constant temperature (20–25°C with 60 ± 5% humidity) with a light cycle of 12 h light/12 h dark and provided with food and water. This study was performed according to the guidelines set by the National Institutes of Health and Institutional Guidelines and was approved by the Ethics Committee of Nankai University (Approval No. 2021-SYDWLL-000038).

### Induction of Liver Fibrosis Mouse Model

We established a liver fibrosis mouse model via intraperitoneal injection of CCl_4_, which was consistent with previous study (Wang et al., 2020). Briefly, CCl_4_ was diluted with olive oil at a concentration of 2 ml/kg body weight and administered via intraperitoneal infusion twice per week over a six-week period.

### Animal Grouping and Dosing Regimen

Thirty mice were used to induce liver fibrosis model. Seven mice died in the first 2 weeks after CCl_4_ injection. After 6 weeks of CCl_4_ injection, three mice were selected randomly and sacrificed. HE staining of liver was used to validate that the liver fibrosis model has been successfully induced. 20 mice were randomly divided into model group and geniposide group. The mice in the control group were intraperitoneally injected with olive oil (2 ml/kg body weight) twice per week for 6 weeks. Mice in the geniposide group were administered geniposide (50 mg/kg daily) via gavage for 4 weeks (Ma et al., 2018; Lin et al., 2019). The mice in the control and model groups were administered an equal amount of saline instead of geniposide. The mice were euthanized after 4 weeks of geniposide treatment, after which their livers were removed and weighed to obtain the liver index. The liver index (%) was calculated according to the following formula: liver weight (g)/body weight (g) × 100%.

### Biochemistry Determination

Once the mice were euthanized, their blood was collected and centrifuged at 3,000 rpm for 15 min to obtain the serum. Liver function was evaluated by assessing the activities of hepatic enzymes in the serum, including ALT and AST. The AST and ALT activities were measured by commercial assay kits according to the manufacturer’s instructions.

### Histopathology

After they were euthanized, the livers of three groups were isolated and immersed in 10% formalin. Liver tissues were dehydrated and paraffin-embedded. The tissues were cut into 5 μm thick strips, stained with hematoxylin and eosin (H&E), and subjected to Masson and Sirius Red staining. The severity of pathological changes in H&E staining were determined through a histological liver fibrosis score based on a previous studies (Klopfleisch, 2013; Aldahmash and El Nagar, 2016). Scores ranged from 0 to 12 (total score), which represents the sum of scores from 0 to 4 for severity of swelling, inflammation, apoptotic cells and fibrosis (Table 1). The relative collagenous fiber areas in Masson and Sirius Red staining were quantified using integrated optical density (IOD) with the Image-Pro Plus 6.0 software. The positive area (%) was calculated according to the following formula: IOD/sum area × 100%.

**TABLE 1:** Liver pathology score.

Feature score	Score	Description
Ballooning degeneration	0	None
	1	Minimal
	2	Mild
	3	Moderate
	4	Severe
Inflammation	0	None
	1	1 inflammatory per field of view
	2	2–4 inflammatory per field of view
	3	>4 inflammatory per field of view
Apoptotic cells	0	None
	1	Few
Fibrosis	0	None
	1	Portal/sinusoidal minimal fibrosis
	2	Portal/sinusoidal mild fibrosis
	3	Bridging fibrosis
	4	Cirrhosis

Scores ranged from 0 to 12 (total score), which represents the sum of scores from 0 to 4 for severity of swelling, inflammation, apoptotic cells and fibrosis.

### Immunostaining

Immunohistochemistry detection of α-SMA in livers was performed on the paraffin for each group. The positive expressed area (%) of α-SMA in liver was analyzed using Image-Pro Plus 6.0 software.

### Assessment of the Oxidative Stress Parameters

Homogenates with 900 µL normal saline for every 0.1 g liver tissue were homogenized via ultrasonic trituration on ice. The protein concentration in liver homogenates was quantified by BCA. The homogenates were centrifuged (3,000 rpm, 15 min) and the supernatant was collected to detect SOD, GSH-Px activities and MDA level using relative commercial kits.

### Determination of Inflammatory Factors in Liver Tissue by Enzyme-Linked Immunosorbent Assay

After 4 weeks of geniposide treatment, the levels of IL-6, IL-1β, and TNF-α in liver tissue homogenates were analyzed using ELISA kits according to the manufacturer’s instructions. The total protein concentration in the liver tissue homogenate was quantified by a BCA protein quantitative kit assay, according to the manufacturer’s instructions. Cytokine concentrations in liver homogenates were assessed according to the following formula: concentration of cytokines in the homogenate/total protein (pg/mg).

### TUNEL Staining

The paraffin sections of the liver tissue were stained using the TUNEL assay kit according to the manufacturer’s instructions. The apoptosis was observed under fluorescence microscope after rinsed with PBS. The ratio of apoptotic cells to the total cells was quantified by Image-pro Plus 6.0 software based on IOD.

### Western Blot

The liver tissues were homogenized and lysed using RIPA lysis buffer to extract the proteins. A BCA Protein Assay Kit was used to measure the total protein concentration. The proteins were separated on an 8–12% sodium dodecyl sulfate polyacrylamide gel electrophoresis and transferred to a polyvinylidene difluoride (PVDF) membrane (Bio-Rad) via electroblotting. The membranes were blocked for 2 h with 5% non-fat milk powder in 1 × TBST at room temperature, after which they were incubated with the primary antibodies (rabbit anti-Bcl-2, 1:800; rabbit anti-Bax, 1:10,000; rabbit anti-Caspase 3, 1:500; rabbit anti- Caspase 9, 1:1,000; and rabbit anti-β-Actin, 1:4,000) overnight at 4°C. After three TBST washes, the membranes were incubated with Goat anti-rabbit IgG (1:10,000) secondary antibodies at room temperature for 2 h. Blotting was observed using ECL. The gray value was quantitatively analyzed using ImageJ software.

### Serum Untargeted Metabolomics Study

We obtained serum samples from the control, model, and geniposide groups to analyze the metabolomics at the end of 4 weeks of geniposide treatment. Differential metabolite screening in the three groups was performed using liquid chromatography-mass spectrometry (LC-MS). Details of the sample preparation and data analysis are described below (Cui et al., 2020).

### Sample Preparation

We added 10 μL of 2-chloro-l-phenylalanine and 10 μL of Lyso PC 17:0 (concentrations were 0.3 and 0.01 mg/ml, dissolved with methanol) to 100 μL of the serum samples.

The serum samples were mixed with 300 μL of methanol:chloroform (2:1, v/v) and centrifuged for 1 min. The mixture was then treated in an ice-water bath for subsequent ultrasonic extraction, after which they were incubated at −20 °C for 1 h to precipitate the proteins. They were centrifuged at 12,000 rpm for 10 min at 4°C, after which 300 μL of the upper solution dried and redissolved with 100 μL [acetonitrile water (1:1)]. The samples were then placed in a 200 μL lined tube for LC-MS analysis. In order to evaluate the stability and repeatability of the system during the sample collection process, all samples were mixed with the same volume to ensure quality control (QC).

### Liquid Chromatography-Mass Spectrometry

Chromatographic separation was performed on a U3000 Ultra Performance Liquid Mass Spectrometer from Thermo Scientific, after which the metabolic profile of each serum sample was analyzed. ACQUITY UPLC HSS T3 columns (2.1*100 mm, 1.8 μm) were used for analysis, in positive and negative modes. The mobile phase was comprised of mobile phase A (0.1% formic acid) and mobile phase B (acetonitrile). The flow rate was constant at 0.3 ml/min, the injection volume was 1.0 µL, and the column temperature was 45°C. Separation was conducted as follows: 0 min, 80% A, 20%B; 2 min, 70% A, 30%B; 5 min, 55% A, 45%B; 6.5 min, 40% A, 60%B; 12 min, 20% A, 80%B; 14 min, 0% A, 100%B; 16 min, 0% A, 100%B; 16.1 min, 80% A, 20%B; 18 min, 80% A, 20%B.

Mass spectrometry (MS) was performed using positive and negative ion mode electrospray ionization (ESI) techniques for MS scanning, with a scanning range from 50 m/z to 1,500 m/z and a resolution of 70,000. The positive and negative ion source voltages were 3.7 and 3.5 kV. The capillary heating temperature of both ions was set to 320°C. The sheath gas pressure and auxiliary gas pressure were 30 and 10 psi, respectively (temperature: 300°C; automatic gain control target: 1 × 10^6^; maximum isolation time: 50 ms). During the analysis, every six samples was tested for quality control to ensure the accuracy of the data.

### Data Processing and Analysis

Progenesis QI software (2.1, Nonlinear Dynamics, Newcastle, United Kingdom) was used for data analysis. Metabolites were identified using the Human Metabolome Database (HMDB, www.hmdb.ca) and local databases. Metabolites with coefficients of variation (CV) less than 30% in the QC samples were kept for the following analysis. Due to large differences in the concentrations of different metabolites, datasets were normalized to avoid masking the signals of metabolites with concentrations that were too high or too low. The principal component analysis (PCA) and orthogonal partial least squares discriminant analysis (OPLS-DA) were performed using SIMCA software (14.1, Umetrics) to visualize the results of the metabolite data results. The quality of the models was evaluated according to their respective R^2^ and Q^2^ values. PCA was used to represent between-group differences and within-group variation. Variable Importance in the Projection (VIP) with a threshold >1 from the OPLS-DA model and *p*-values (*p* < 0.05) from the t-test were used to search differentially expressed metabolites. The normalized peak areas of all metabolites were expressed as mean ± standard (mean ± SD) deviation and analyzed using a two-tailed Student’s t-test.

Metabolites with fold changes higher than 1.25 or less than 0.80 were selected for metabolic pathway analysis. The MetaboAnalyst 5.0 software (www.metaboanalyst.ca) was used to analyze the metabolic pathways of the differential metabolites between two groups of samples (control and model; model and geniposide). The KEGG (www.kegg.jp) database was used for pathway analysis.

### Statistical Analysis

All experimental data were expressed as the mean ± SD, and statistical differences between the experimental groups were evaluated using one-way ANOVA via SPSS software (version 20.0, United States). *p*-values were generated by the log-rank test, and *p* < 0.05 was considered statistically significant.

## Results

### Effects of Geniposide on CCl_4_-Induced Liver Fibrosis Model Mice

**FIGURE 2:**
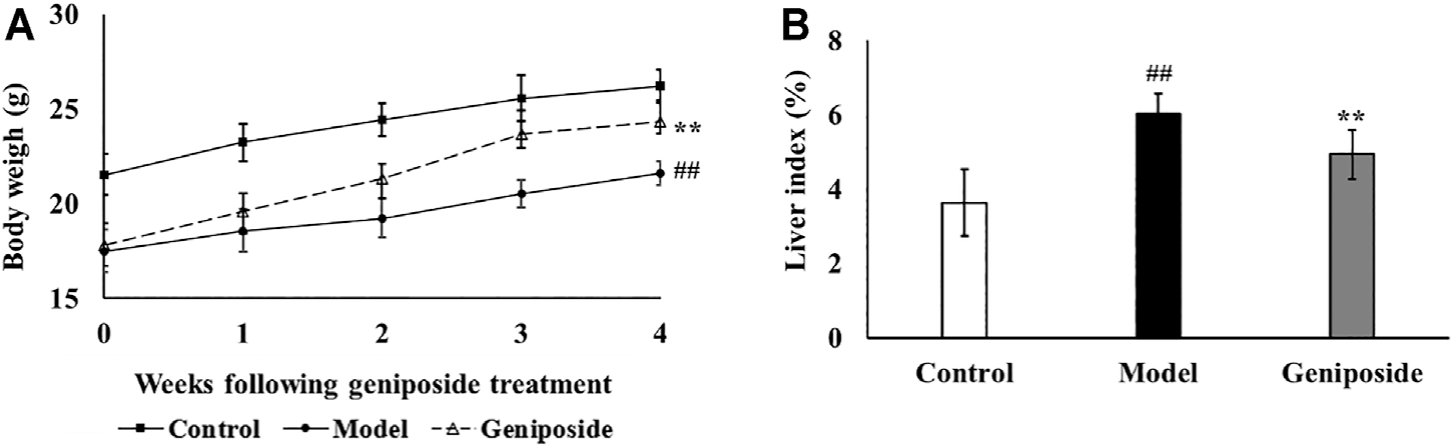
Geniposide treatment increases body weight and liver index. **(A)** Body weight of 3 groups. **(B)** Liver index of 3 groups. Control, model, and geniposide groups (*n* = 10 per group). ^##^ *p* < 0.01, Model group *vs.* Control group; ^∗^ *p* < 0.05 and ^∗∗^ *p* < 0.01, Geniposide group *vs.* Model group.

After 4 weeks of geniposide administration, the efficacy of the treatment was evaluated by measuring the body weight, liver index, hepatic enzyme activities of ALT and AST, and pathological changes in liver. As shown in Figure 2A, the body weight in the model group was significantly lower than that in the control group (*p* < 0.01). After 4 weeks of geniposide treatment, body weight increased in liver fibrosis model mice (*p* < 0.01). CCl_4_ treatment resulted in significant (*p* < 0.01) increases in the liver index compared to the control group. Geniposide treatment decreased the liver index in the model group (*p* < 0.05, Figure 2B). Compared to the control group, AST and ALT activities in the model group significantly increased. Geniposide treatment reduced them (*p* < 0.01, Table 2) of liver fibrosis model mice.

**TABLE 2:** Effects of geniposide ALT and AST levels in serum.

Group	AST (U/L)	ALT (U/L)
Control	102.79 ± 16.34	33.41 ± 8.57
Model	203.13 ± 36.75^##^	82.26 ± 12.48^##^
Geniposide	149.15 ± 22.96^**^	51.95 ± 10.81^**^

Control, model, and geniposide treated groups (*n* = 10 per group). Data are presented as mean ± SD. ^##^ *p* < 0.01, Model group *vs.* Control group; ^∗∗^ *p* < 0.01, Geniposide group *vs.* Model group.

**FIGURE 3:**
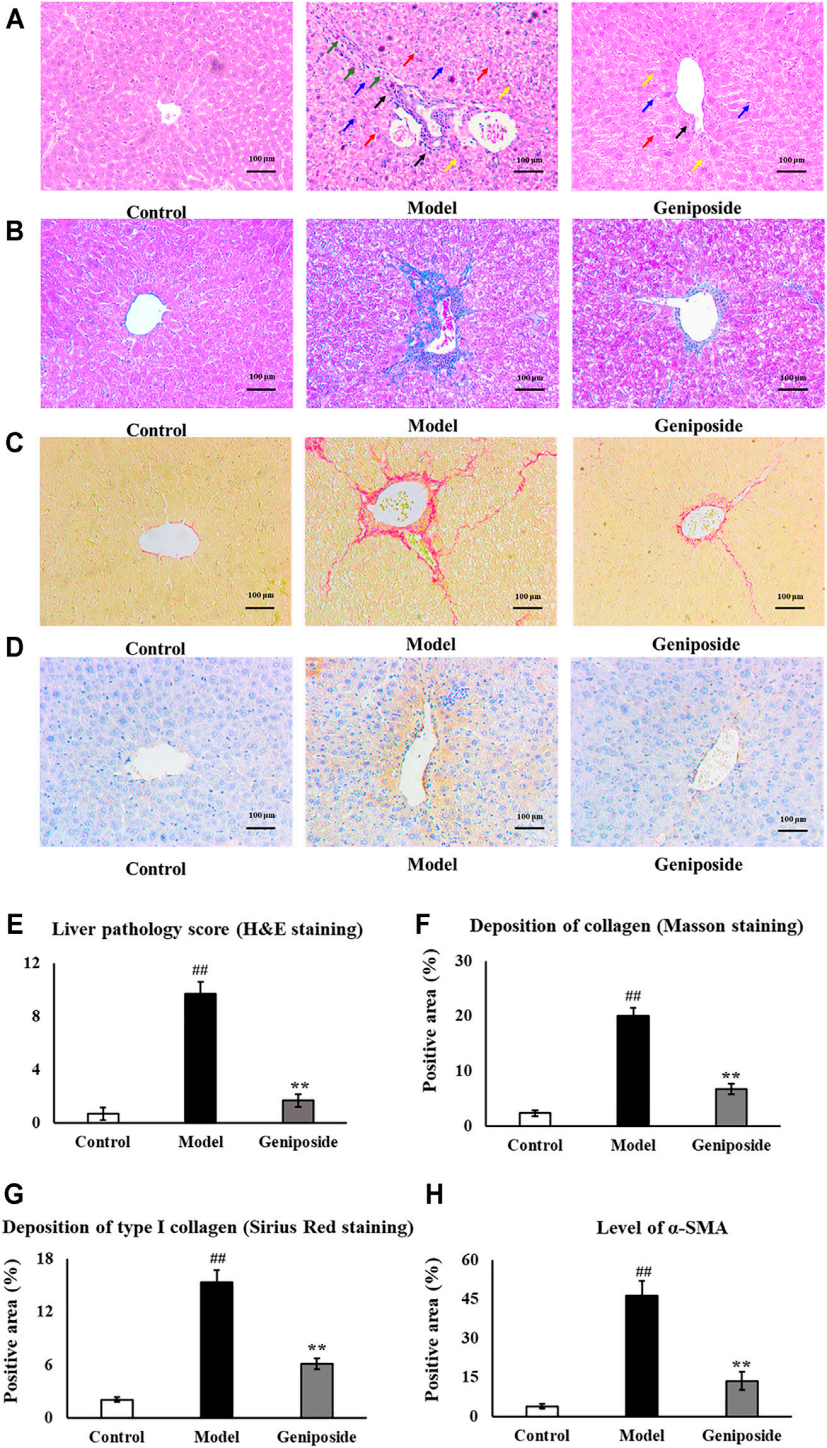
Evaluation of liver histopathology. **(A,E)** H&E staining and liver pathology score showed that geniposide treatment improved pathological changes in the liver. Black arrows indicate infiltration of inflammatory cells. Yellow arrows indicate structural disorder of the hepatic cord. Red arrows indicate swelling of the liver cells. Blue arrows indicate necrosis of the liver cells. Green arrows indicate fibrosis. **(B,F)** Masson staining showed that geniposide treatment could reduce collagen deposition in the liver. **(C,G)** Sirius Red staining showed that geniposide treatment reduced liver type Ⅰ collagen deposition. **(D H)** Immunostaining showed that geniposide treatment could decrease α-SMA levels in the liver (100×). Control, model, and geniposide groups (*n* = 10 per group). Data are presented as mean ± SD. ^##^ *p* < 0.01, Model group *vs.* Control group; ^∗∗^ *p* < 0.01, Geniposide group *vs.* Model group.

Pathological changes in the liver following geniposide treatment were measured by H&E, Masson and Sirius Red staining, and immunostaining. H&E staining and liver pathology score showed that the hepatocytes in the control group were arranged radially along the central vein, while the liver structure was clearly visible. The model group’s livers exhibited hepatic cord disorder, hepatocellular swelled and necrosis, nuclear shrinkage and disappearance, inflammatory cell infiltration and fibrosis appeared, while geniposide treatment improves morphological damage in hepatic tissues (Figures 3A,E).

Masson staining showed an increased overall collagen deposition and fibrotic lesions in liver fibrosis model mice. Sirius Red staining showed that type Ⅰ collagen significantly increased in liver fibrosis model mice. Following geniposide treatment, we observed significant reductions in collagen deposition and fibrotic lesions; the only area with visible collagen deposition was the portal area (*p* < 0.01, Figures 2B,C,F,G).

Immunostaining demonstrated that the expression of α-SMA in the model group was higher than that in the control group (*p* < 0.01, Figures 2D,H). Geniposide treatment could reduce α-SMA levels compared to the model group (*p* < 0.01, Figures 2H, 3D).

### Effects of Geniposide on Inflammation and Oxidative Stress in Liver Fibrosis Model Mice

**FIGURE 4:**
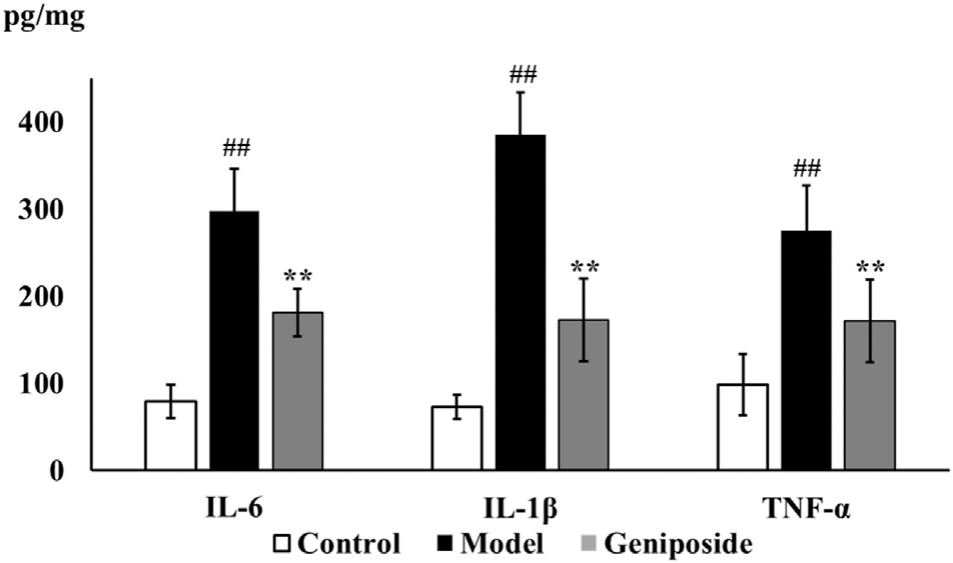
Geniposide treatment improved inflammatory response in liver fibrosis model mice. ELISA test showed that geniposide treatment decreased the levels of IL-6, IL-1β, and TNF-α in liver tissue homogenate. Control, model, and geniposide groups (*n* = 10 per group). ^##^ *p* < 0.01, Model group *vs.* Control group; ^∗^ *p* < 0.05 and ^∗∗^ *p* < 0.01, Geniposide group *vs.* Model group.

We analyzed the impacts of geniposide on oxidative stress by detecting MDA levels and the activities of SOD and GSH-Px in liver tissue homogenate. Our results demonstrated that SOD (*p* < 0.01) and GSH-Px activities (*p* < 0.01) decreased and that MDA levels (*p* < 0.05) increased in the model group compared to the control group, while SOD (*p* < 0.05) and GSH-Px activities (*p* < 0.05) increased and MDA levels (*p* < 0.05) decreased in the geniposide group compared to the model group (Table 3). The anti-inflammatory effect was evaluated by detecting IL-6, IL-1β, and TNF-α levels in liver tissue homogenate using ELISA. Our results demonstrated that IL-6, IL-1β, and TNF-α levels increased in the model group compared to the control group (*p* < 0.01), and that geniposide treatment decreased IL-6, IL-1β (*p* < 0.01), and TNF-α (*p* < 0.05) levels in the liver tissue homogenate compared to the model group (Figure 4).

**TABLE 3:** SOD, GSH-Px activities, and MDA levels in liver homogenates after geniposide treatment.

Group	SOD (U/mg prot)	GSH-Px (U/mg prot)	MDA (nmol/mg prot)
Control	30.44 ± 3.29	265.76 ± 36.78	2.44 ± 0.64
Model	23.90 ± 1.24^##^	139.41 ± 45.49^##^	4.55 ± 1.26^##^
Geniposide	29.15 ± 4.75^**^	225.29 ± 43.09^**^	2.76 ± 0.47^**^

Control, model, and geniposide groups (*n* = 10 per group). Data are presented as mean ± SD. #*p* < 0.05 and ^##^ *p* < 0.01, Model group *vs.* Control group; ^∗^ *p* < 0.05, Geniposide group *vs.* Model group.

### Effects of Geniposide on the Apoptosis of Cells in Liver Fibrosis Model Mice

**FIGURE 5:**
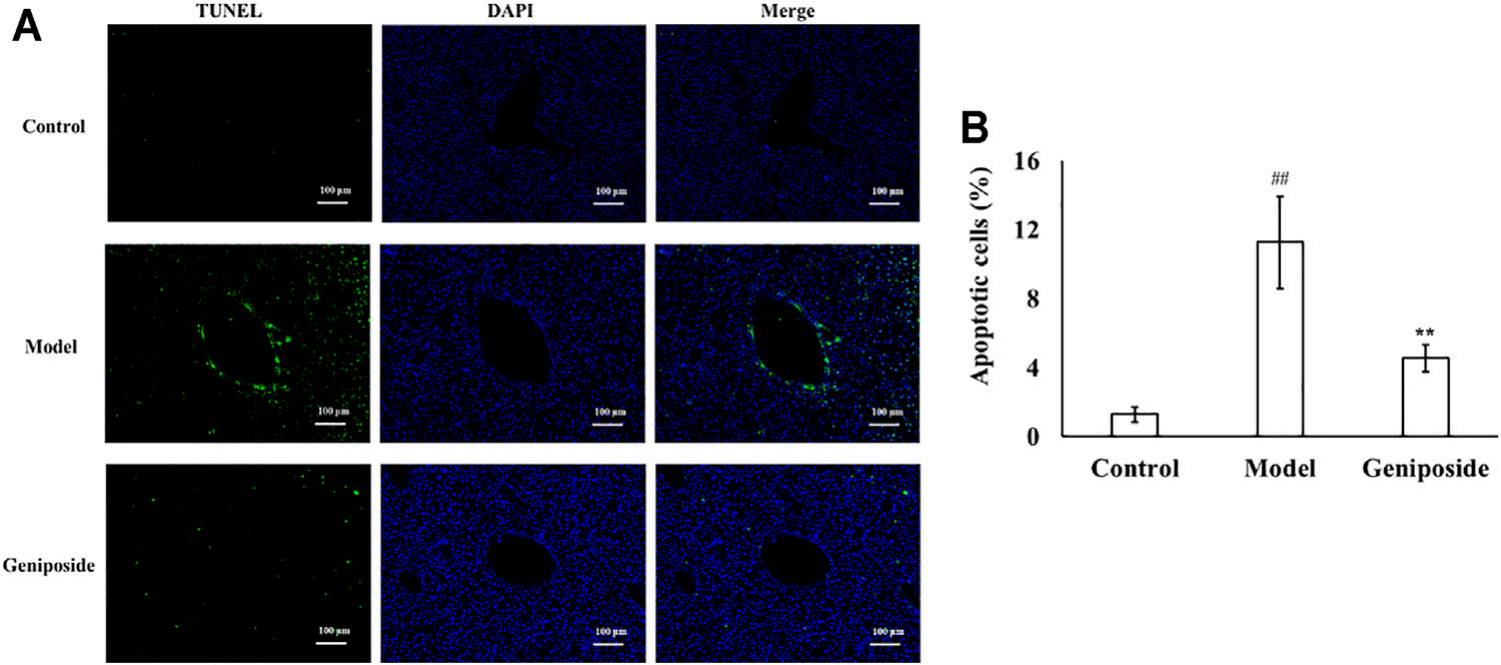
Geniposide treatment improved apoptosis in liver fibrosis model mice. **(A)** Changes of TUNEL staining. **(B)** Changes of quantitative analysis of apoptotic cells. **(A,B)** TUNEL staining indicated that geniposide treatment reduced the number of apoptotic cells in a liver section. Control, model, and geniposide groups (*n* = 10 per group). Data are presented as mean ± SD. ^##^ *p* < 0.01, Model group *vs.* Control group; ^∗∗^ *p* < 0.01, Geniposide group *vs.* Model group.

TUNEL staining showed that there were almost no apoptotic cells in the liver tissue of the control group, while the numbers of apoptotic cells in the model group increased. The apoptotic cells decreased more after geniposide treatment compared to the model group. Image analysis of the apoptosis index of liver tissue with fibrosis in different groups is displayed in Figure 5.

### Effects of Geniposid on Apoptosis-Related Factors in Liver Fibrosis Model Mice

**FIGURE 6:**
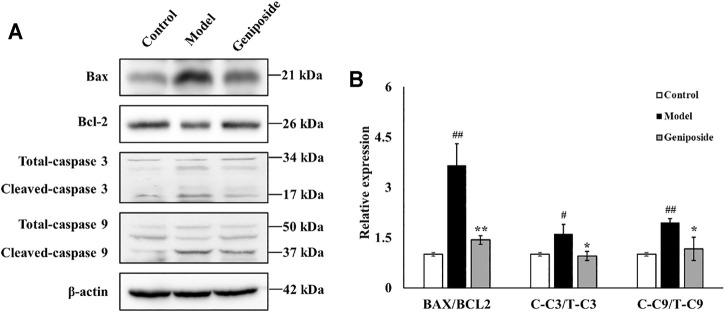
Geniposide treatment regulated BAX, Bcl-2, Caspase 3, and Caspase 9 in liver fibrosis model mice. **(A,B)** Changes of the protein expression of BAX, Bcl-2, Caspase 3, Caspase 9, and β-actin in liver tissue. Geniposide treatment reversed the relative expression of the BAX, Bcl-2, Caspase 3, and Caspase 9 in model mice liver tissue. Control, model, and geniposide groups (*n* = 3 per group). #*p* < 0.05, Model group *vs.* Control group; ##*p* < 0.01, Model group *vs.* Control group; ∗*p* < 0.05 and ∗∗*p* < 0.01, Geniposide group *vs.* Model group.

We measured the expressions of Bax, Bcl-2, Caspase 3, and Caspase 9, which are all involved in apoptosis, to determine the anti-apoptotic activities of geniposide in CCl_4_-induced liver fibrosis. We found that the expressions of Bax, Caspase 3, and Caspase 9 were all elevated and that the expression of Bcl-2 decreased in the model group compared with the control group (for Caspase 3, *p* < 0.05; for Bax, Bcl-2 and Caspase 9, *p* < 0.01; Figure 4). Geniposide treatment decreased the expressions of Bax, Caspase 3, and Caspase 9 and increased the expression of Bcl-2 compared with the model group (for Caspase 3 and Caspase 9, *p* < 0.05; for Bax and Bcl-2, *p* < 0.01; Figure 6).

### Multivariate Analysis of Serum Metabolomics

**FIGURE 7:**
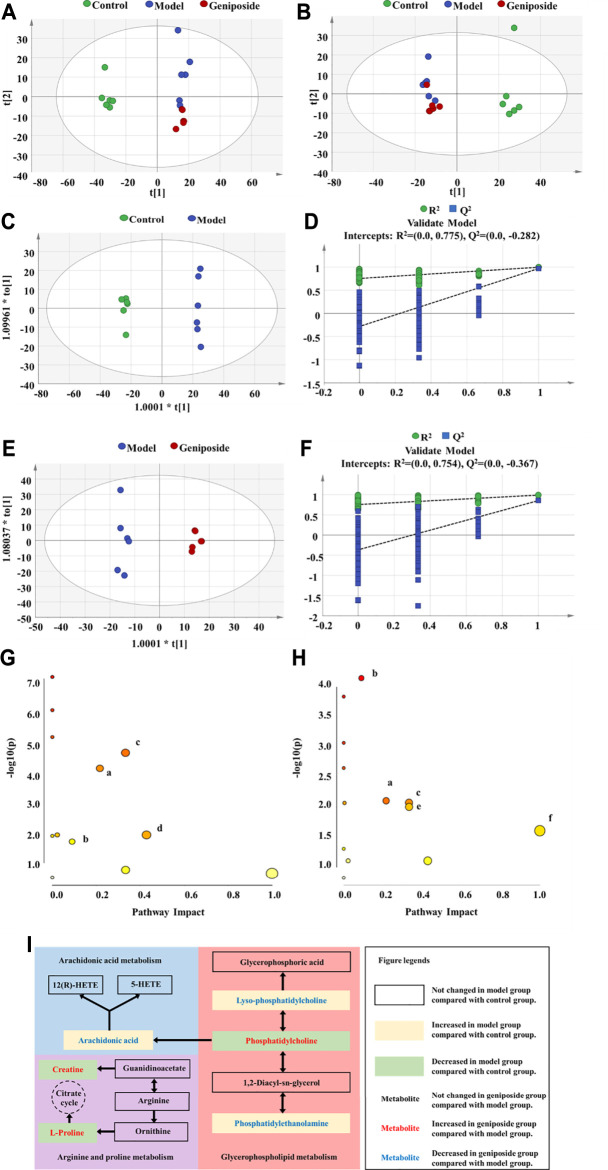
Serum metabolism analysis of geniposide in liver fibrosis. **(A)** PCA score plot among control, model, and geniposide groups in positive mode. **(B)** PCA score plot among control, model, and geniposide groups in negative mode. **(C,D)** OPLS-DA score plot among control and model groups and the corresponding coefficient of loading plots. **(E,F)** OPLS-DA score plot among model and geniposide groups and the corresponding coefficient of loading plots. **(G)** Pathway analysis between control and model groups. **(H)** Pathway analysis between model and geniposide groups. (a) Glycerophospholipid metabolism, (b) Arginine and proline metabolism, (c) AA metabolism, (d) Taurine and hypotaurine metabolism, (e) alpha-Linolenic acid metabolism, and (f) Linoleic acid metabolism. Control, model, and geniposide treated groups (*n* = 6 per group). #: *p* < 0.05 and ##*p* < 0.01, Model group vs. Control group; ∗*p* < 0.05 and ∗∗*p* < 0.01, Geniposide group vs. Model group.

Using untargeted metabolomics, we compared the changes of metabolites in the serum of all three groups. The metabolite profiles were analyzed via PCA and OPLS-DA. Our results demonstrated that there were significant differences between the control, model, and geniposide groups. The PCA model had an R^2^ value of 0.641 and a Q^2^ value of 0.366 (Figures 7A,B) and the OPLS-DA model had an R^2^ value of 0.999 and a Q^2^ value of 0.970 (Figures 7C–F).

### Identification and Pathway Analysis of Differential Metabolites

A total of 24 differential metabolites were considered to be differential metabolites with a VIP >1 and *p* < 0.05 (fold change (FC) greater than 1.2 or less than 0.8) in the model group *vs.* control group or geniposide group *vs.* model group. These differential metabolites are listed in Table 4. Compared with the control group, levels of Decanoylcarnitine, Hexacosanoic acid, L-Palmitoylc, LysoPC(18:0), PE [18:0/18:1 (9Z)], PE (P-16:0e/0:0), Myristic acid, Arachidonic acid (AA), Oleic acid, Eicosadienoic acid, and Adrenic acid all increased in the model group, while levels of Hexanoylcarnitine, L-Octanoylcarnitine, Pentacosanoic acid, Creatine, L-Carnitine, Taurine, PC [18:0/18:2 (9Z,12Z)], Docosahexaenoic acid, L-Proline, Alpha-Linolenic acid, Linoleic acid, Nervonic acid, and PC (16:1 (9Z)/14:0) all decreased in the model group. When the geniposide group was compared with the model group, the levels of Decanoylcarnitine, L-Palmitoylcarnitine, LysoPC(18:0), PE [18:0/18:1 (9Z)], PE (P-16:0e/0:0), AA, Oleic acid, and Eicosadienoic acid all decreased, while levels of Hexanoylcarnitine, Creatine, Taurine, PC [18:0/18:2 (9Z,12Z)], Docosahexaenoic acid, L-Proline, Alpha-Linolenic acid, Linoleic acid, Nervonic acid and PC (16:1 (9Z)/14:0) increased (Table 4). Differential metabolites were used for pathway analysis. These products were then used to select pathways with impact values greater than 0.05 and *p*-values less than 0.05. Four pathways were screened out that varied between the control and model groups: Taurine and hypotaurine metabolism, Glycerophospholipid metabolism, Arginine and proline metabolism, and AA metabolism (Figure 7G). Five pathways were screened out that varied between the model and geniposide groups: Glycerophospholipid metabolism, Arginine and proline metabolism, AA metabolism, alpha-Linolenic acid metabolism, and Linoleic acid metabolism. Therefore, the common pathways between the control and model groups and the model and geniposide groups were considered significant pathways: Glycerophospholipid metabolism, Arginine and proline metabolism, and AA metabolism (Figure 7H). The relationships between these pathways are displayed in Figure 7I.

**TABLE 4:** Summary of the differential metabolites identified in the serum of three Groups.

No.	Rt (min)	m/z	Formula	Metabolites	VIP	FC		Trend			Pathway
						M vs. C	G vs. M	M vs. C		G vs. M	
1	5.42	260.19	C13H25NO4	Hexanoylcarnitine	1.68	0.21	3.28	↓##	↑**		—
2	6.29	288.22	C15H29NO4	L-Octanoylcarnitine	1.63	0.22	1.27	↓##	↑		—
3	6.83	316.25	C17H33NO4	Decanoylcarnitine	1.60	2.16	0.70	↑##	↓**		—
4	13.95	395.39	C26H52O2	Hexacosanoic acid	1.08	5.53	0.79	↑##	↓		—
5	13.17	381.37	C25H50O2	Pentacosanoic acid	1.07	0.53	0.79	↓##	↓		—
6	7.70	400.34	C23H45NO4	L-Palmitoylcarnitine	1.07	2.09	0.76	↑##	↓*		—
7	0.90	132.08	C4H9N3O2	Creatine	1.07	0.68	1.32	↓##	↑**		b
8	0.86	162.11	C7H15NO3	L-Carnitine	1.06	0.70	1.27	↓##	↑		—
9	0.86	126.02	C2H7NO3S	Taurine	1.06	0.66	1.29	↓##	↑		d
10	9.10	524.37	C26H54NO7P	LysoPC(18:0)	1.03	1.26	0.79	↑##	↓**		a
11	13.08	786.60	C44H84NO8P	PC (18:0/18:2 (9Z,12Z))	1.04	0.72	1.33	↓#	↑*		a, c, e, f
12	9.03	327.23	C22H32O2	Docosahexaenoic acid	1.06	0.66	1.34	↓##	↑**		—
13	12.24	746.57	C41H80NO8P	PE (18:0/18:1 (9Z))	1.05	1.68	0.69	↑##	↓**		a
14	0.90	116.07	C5H9NO2	L-Proline	1.06	0.57	1.40	↓##	↑**		b
15	8.88	277.22	C18H30O2	Alpha-Linolenic acid	1.07	0.60	1.57	↓#	↑**		—
16	9.13	279.23	C18H32O2	Linoleic acid	1.08	0.76	1.29	↓#	↑*		f
17	8.80	438.30	C21H44NO6P	PE (P-16:0e/0:0)	1.09	1.73	0.73	↑##	↓**		—
18	11.58	365.34	C24H46O2	Nervonic acid	1.08	0.78	1.55	↓##	↑**		—
19	8.85	227.20	C14H28O2	Myristic acid	1.09	1.91	0.79	↑##	↓		—
20	9.09	303.23	C20H32O2	Arachidonic acid	1.11	2.09	0.77	↑##	↓**		c
21	9.45	281.25	C18H34O2	Oleic acid	1.44	1.44	0.73	↑##	↓**		e
22	11.03	704.52	C38H74NO8P	PC (16:1 (9Z)/14:0)	1.43	0.58	1.29	↓#	↑*		—
23	9.62	307.26	C20H36O2	Eicosadienoic acid	1.65	4.27	0.54	↑##	↓**		—
24	9.47	331.26	C22H36O2	Adrenic acid	1.65	4.58	0.74	↑##	↓		—

Control, model, and geniposide groups (*n* = 6 per group). ^#^ *p* < 0.05 and ^##^ *p* < 0.01, Model group *vs.* Control group; ^∗^ *p* < 0.05 and ^∗∗^ *p* < 0.01, Geniposide group *vs.* Model group.

(a) Glycerophospholipid metabolism, (b) Arginine and proline metabolism, (c) AA metabolism, (d) Taurine and hypotaurine metabolism, (e) alpha-Linolenic acid metabolism, and (f) Linoleic acid metabolism.

## Discussion

CCl_4_ is a commonly used to induce liver injury and liver fibrosis models (Zhu et al., 2019; Chen et al., 2021). Previous study showed that Swiss albino mice developed chronic liver injury after receiving oral treatment of CCl_4_ (1.5 ml/kg) once daily for 8 weeks (Zhang et al., 2020). Intraperitoneal injection of CCl_4_ (1 ml/kg) three times per week for 8 weeks could induce severe oxidative stress in Wistar rats (Goodla et al., 2019). Based on our previous study, we treated mice with intraperitoneal injection of CCl_4_ (2 ml/kg) twice per week over a six-week period (Wang et al., 2020). Compared with the control group, the liver index, serum ALT, and AST levels of the model mice all increased. Additionally, pathological analysis of the liver tissue found hepatocyte degeneration, inflammatory infiltration, fibrous connective tissue hyperplasia, damaged liver lobule structure, and the generation of a fibrous septum. This indicates that the model was successful. After being treated with geniposide, the ALT and AST values in the serum and the liver index decreased, resulting in improved pathological changes in the liver. This suggests that geniposide can ameliorate liver fibrosis induced by CCl_4_.

Oxidative stress, inflammatory response and apoptosis are the major pathological characteristics during the progression of liver fibrosis (Zhu et al., 2020). We further evaluated the effects of geniposide on oxidative stress, inflammatory response and apoptosis in liver fibrosis mice. Our results found that geniposide could increase SOD and GSH-Px activities and decrease MDA content in the liver, indicating that geniposide could alleviate the oxidative stress reaction in model mice with liver fibrosis. Increased oxidative stress levels are a sign of the presence and development of liver fibrosis. In hepatocytes, CCl_4_ could be transformed into active trichloromethylradical (CCl_3_) under the catalyzation of cytochrome oxidase P450. Subsequently, CCl_3_ could react with O_2_ and generate more free radicals, resulting in lipid peroxidation and further damage to hepatocytes (Hermenean et al., 2012). MDA is an intermediate product of lipid peroxidation degradation and can seriously impair the composition, structure, and function of cells. MDA content can reflect the rate of lipid peroxidation in the body, and is an indicator of free radical content, indirectly indicating levels of cell damage (Shi et al., 2020). SOD and GSH-Px are anti-oxidative enzymes that demonstrate anti-oxidative strength and can protect liver cells from oxygen free radicals (El-Din et al., 2014). SOD is a vital scavenger of oxygen free radicals in the enzyme defense system, and can specifically scavenge superoxide anions and protect cells from damage (Forbes-Hernández et al., 2017). GSH-Px is a significant peroxide decomposing enzyme found throughout the body that eliminates free radicals such as hydrogen peroxide and prevents the formation of lipid peroxide. GSH-Px can catalyze the reaction between lipid peroxide and reduced glutathione to form oxidized glutathione, and plays a role in preserving the integrity of the structure and function of cell membranes (Lin et al., 2015).

ELISA experiments demonstrated that geniposide could reduce the contents of IL-6, IL-1β, and TNF-α in model mice and alleviate inflammatory reactions. CCl_4_ could cause hepatocellular damage and subsequently activate Kupffer cells (Han et al., 2016). Activated Kupffer cells could produce inflammatory cytokines such as IL-6, IL1β and TNF-α. (Wang et al., 2019). These inflammatory factors contribute to the progression of liver fibrosis, while TNF-α can stimulate collagen synthesis and IL-6 and IL-1β can activate hepatic stellate cells and directly stimulate collagen secretion (Lee et al., 2014; Sun et al., 2018).

TUNEL staining and Western blot analysis were used to assess the apoptosis of liver tissues. TUNEL results demonstrated that geniposide could improve hepatocyte apoptosis in model mice. Western blot analysis demonstrated that the expressions of Bax, Caspase 3 and Caspase 9 all decreased and that the expression of Bcl-2 increased following geniposide treatment. During the development of liver fibrosis, the liver tissue is subjected to apoptosis (Jaeschke, 2006).

Excessive free radicals induced by CCl_4_ could lead to the released of Cytc and then trigger the apoptosis of hepatocytes in liver (Ki et al., 2013; Mekky et al., 2016). Apoptosis is a process regulated by multiple factors, including Bax, Bcl-2, and the Caspase family (Tian et al., 2015). Bax and Bcl-2 are a pair of homologous regulatory factors with antagonistic effects. After forming a dimer, they induce apoptosis by changing the permeability of the outer membrane of the mitochondria (Moldoveanu et al., 2014; Hwang-Bo et al., 2019). Bax is a significant pro-apoptotic factor and participates in the mitochondrial apoptosis pathway. Bax forms holes in the outer membrane of the mitochondrial after activation, resulting in the loss of membrane integrity (Tait and Green, 2010). The anti-apoptotic factor Bcl-2 can inhibit Bax function, while the ratio between Bax and Bcl-2 determines the strength of their apoptosis-inducing effect. The Caspase family is another key regulator of the apoptosis pathway, and can also promote apoptosis (Zhao et al., 2018). In this family, Caspase-9 is upstream of the cascade reaction and belongs to the subclass of apoptosis initiation. Caspase-9 participates in the downstream initiation of Caspase-3 protease, which can directly induce apoptosis (Häcker and Paschen, 2007; Huang et al., 2021). As Bax expression in the Bax/Bcl-2 dimer increases, it will activate Caspase-9, and Caspase-9 will activate Caspase-3 *via* enzyme digestion. This will promote the cleavage of Caspase-3, beginning the apoptosis cascade reaction and resulting in apoptosis (Zhu et al., 2012; Perumal et al., 2018).

According to untargeted metabolomics, the metabolic profiles of the control group, the model group, and the geniposide group all differed, suggesting that geniposide affects the metabolic profiles of model mice with liver fibrosis induced by CCl_4_. Recently, the metabolic regulatory effect of geniposide on liver disease models is becoming a hot spot. *In vivo* study showed that geniposide could regulate the interconversion of pentose and glucuronate and the biosynthesis of primary bile acid in jaundice model mice (Fang et al., 2020). Moreover, integrated miRNAomics, proteomics and metabolomics analysis found that geniposide could upregulate the protein levels of isocitrate dehydrogenase (IDH) 1 and 2 through downregulating the expression of miR-144-5p and then modulate energy metabolism in ethanol-induced apoptosis hepatocyte model *in vitro* (Qiu et al., 2017). Lipid peroxidation induced by CCl_4_ treatment can alter the permeability of the cell membrane, resulting in damage to the intracellular organelle membrane structures, such as mitochondria, endoplasmic reticulum, and Golgi apparatus, etc., which further triggers the metabolic disorders in hepatocytes (Abdou et al., 2019). Untargeted metabolomics were used to analyze the metabolic regulatory effects of geniposide on CCl_4_-induced liver fibrosis model. Our study found that glycerophospholipid metabolism, arginine and proline metabolism, and AA metabolism all changed between the control and model groups and between the model and geniposide groups. These results suggest that geniposide could improve liver fibrosis by regulating glycerophospholipid metabolism, arginine and proline metabolism, and AA metabolism.

### Glycerophospholipid Metabolism

Glycerophospholipid is an important component of biofilm. Its metabolism is involved in many biological processes, such as membrane fusion, endocytosis, and membrane transport. Metabolism disorders related to glycerophospholipid can adversely affect the metabolism of the liver (Xue et al., 2020). The results of our metabolomic analysis indicated that PC decreased and Lyso PC and PE both increased in model mice with liver fibrosis, and that PC levels increased and Lyso PC and PE decreased following geniposide treatment. PC accounts for 40–50% of total glycerophospholipids, is the most abundant glycerophospholipids found in mammalian cells (Zhang et al., 2016). PC can promote collagenase activity, hydrolyze collagenated fibers, and alleviate liver fibrosis (Chen et al., 2016). PC has also demonstrated an anti-oxidative effect. PC can stop active free radicals from attacking the liver cell membrane and decrease stellate cell activity in the liver (Shmarakov et al., 2019). PE accounts for 40% of total glycerophospholipids, and primarily exists in the mitochondrial membrane (Rashid et al., 2020). PE is closely associated with cell proliferation and differentiation, and could trigger apoptosis (Xue et al., 2014). A decrease in the PC/PE ratio can destroy the integrity of the hepatocyte and mitochondrial membrane and affect cell growth and induce apoptosis (Li, et al., 2006). Lyso PC could bind to the G protein-coupled receptor and the Toll-like receptor, promoting macrophage migration, stimulating the production of inflammatory factors, and inducing oxidative stress and apoptosis (Kabarowski et al., 2001; Chang et al., 2008; Kim et al., 2009; Bach et al., 2010; Song et al., 2010; Carneiro et al., 2013). Lyso PC exacerbated inflammation and promoted the development of liver fibrosis (Liu et al., 2020).

### AA Metabolism

AA metabolism is a significant factor in inflammation (Gai et al., 2018; Sonnweber et al., 2018; Melgar-Lesmes et al., 2019). The results of our experiment demonstrated that AA content in the model group increased compared with the control group, a trend that decreased following geniposide treatment. AA metabolites include a series of prostaglandins and leukotrienes, which are highly active inflammatory mediators and can promote the infiltration and activation of inflammatory cells in the liver tissue, leading to the degeneration and necrosis of hepatocytes (Cardoso et al., 2003). AA not only plays a key role in inflammation, but also mediates the production of oxygen free radicals and membrane lipid peroxidation as a lipid medium. AA significantly increases the production of reactive oxygen species, which results in the oxidation of a large number of fatty acids and the formation of lipid peroxides in mitochondria, peroxisomes, endoplasmic reticulum, and other parts of hepatocytes. This can lead to oxidative stress and hepatocyte injury, hepatic stellate cells activation, and collagen deposition in liver (Moreno, 2003; Koek et al., 2011; Fujii and Kawada, 2012). Therefore, the effects of geniposide on inflammation and oxidative stress may be associated with modulating AA metabolism.

### Arginine and Proline Metabolism

The arginine and proline metabolism pathway was closely linked to the progression of liver fibrosis. In model mice with liver fibrosis, we found that L-Proline and creatine in the arginine and proline metabolism pathway significantly increased, but decreased following geniposide treatment. Arginine is the largest nitrogen-supplying amino acid in the human body and is the precursor of proline and creatine (Ge et al., 2020). Proline plays a central role in metabolism, which is closely associated with oxidative stress and apoptosis (Pandhare et al., 2006). Proline can metabolize and produce electrons, and generate reactive oxygen species, leading to a variety of downstream effects, including blocking cell cycles, autophagy, and apoptosis (Selen et al., 2015). Creatine can alleviate liver injuries by inhibiting liver inflammation, oxidative stress, and cell senescence (Deminice et al., 2016; Aljobaily et al., 2020). Creatine can also reduce the consumption of adenosylmethionine and the production of homocysteine in the liver, lowering the damage to homocysteine metabolism in the liver and the accumulation of lipids (Barcelos et al., 2016).

## Conclusion and Future Prospective

In conclusion, our study demonstrated that geniposide could exhibit protective effects on liver fibrosis. This study is the first to screen the differential metabolites of geniposide on CCl4-induced liver fibrosis model. Our results further refine the metabolic regulatory effects of geniposide on liver diseases. The mechanism behind these protective effects could be related to inhibiting oxidative stress, inflammatory response, and apoptosis in the liver and modulating glycophorophospholipid, arginine and proline, and AA metabolism possesses.

## Data Availability

The original contributions presented in the study are included in the article/Supplementary Material, further inquiries can be directed to the corresponding authors.
