# Supplementary material for: Geniposide Ameliorates Liver Fibrosis Through Reducing Oxidative Stress and Inflammatory Respose, Inhibiting Apoptosis and Modulating Overall Metabolism
Source: Front Pharmacol. 2021 Nov 24;12:772635. doi: 10.3389/fphar.2021.772635 (PMC8651620; doi:10.3389/fphar.2021.772635)
Supplement: Supplementary file 1 [file DataSheet1.PDF]

Supplementary Figures

Geniposide treatment regulated BAX, Bcl-2, Caspase 3, and Caspase 9 in liver fibrosis model mice

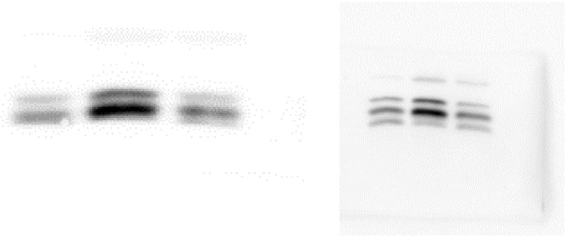

Changes of the protein expression of BAX

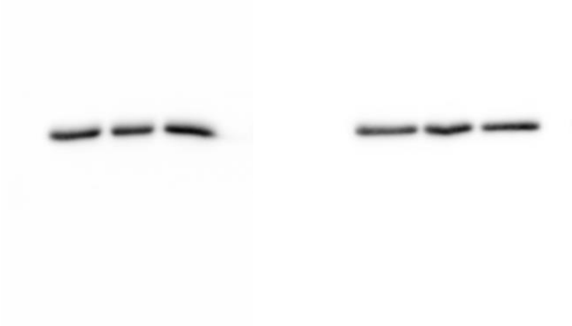

Changes of the protein expression of Bcl-2

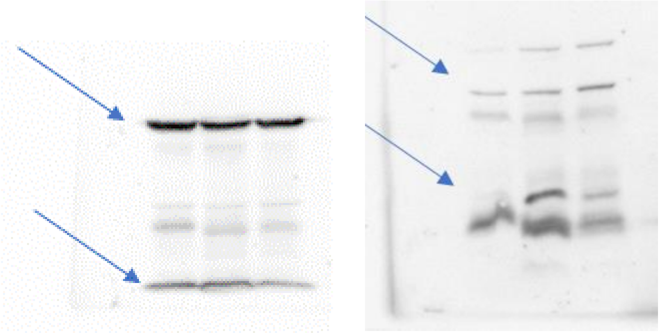

Changes of the protein expression of Caspase 3

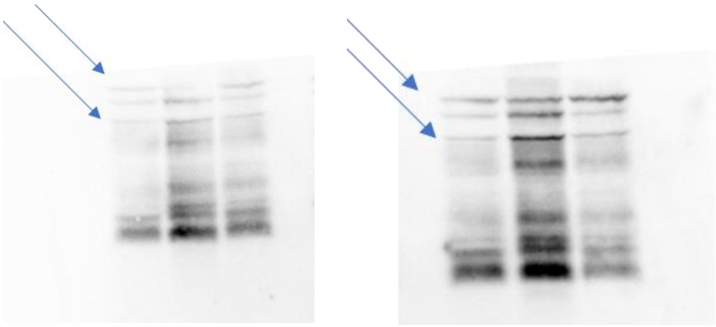

Changes of the protein expression of Caspase 9

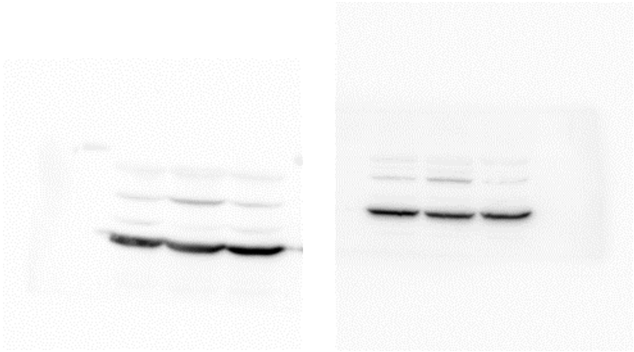

Changes of the protein expression of β-actin
